# Supplementary material for: Plant Growth Promotion Function of Bacillus sp. Strains Isolated from Salt-Pan Rhizosphere and Their Biocontrol Potential against Macrophomina phaseolina
Source: Int J Mol Sci. 2021 Mar 24;22(7):3324. doi: 10.3390/ijms22073324 (PMC8036593; doi:10.3390/ijms22073324)
Supplement: Supplementary file 1 [file ijms-22-03324-s001.pdf]

International Journal of
Molecular Sciences

MDPI

Supplementary Material

Table S1. Preliminary characterization of spore-forming bacteria isolated from the rhizosphere of *J. sabina* plants.

Isolate Code	Colony Color	Colony Morphology	*Anaerobic Growth	pH Range	Temperature Range (°C)
RHFS01	Creamy-white	Rhizoid	++	4–12	15–50
RHFS02	Creamy-white	Irregular	++	4–12	25–50
RHFS03	Creamy-white	Rhizoid	+++	4–12	15–60
RHFS04	Creamy-white	Punctiform	+++	4–12	15–60
RHFS05	Creamy-white	Lobate	+	4–12	15–50
RHFS06	Creamy-white	Irregular	+++	6–12	15–60
RHFS07	Brown	Circular	+++	4–12	15–60
RHFS08	Creamy-white	Filamentous	+	4–12	15–50
RHFS09	Creamy-white	Irregular	+++	2–12	15–50
RHFS10	White	Undulate	++	6–12	15–50
RHFS11	Creamy-white	Irregular	+++	4–12	15–60
RHFS12	Creamy-white	Circular	+++	4–12	15–60
RHFS13	Creamy-white	Circular	+++	2–12	15–60
RHFS14	Creamy-white	Circular	+	4–12	15–50
RHFS15	Creamy-white	Irregular	+++	4–12	15–60
RHFS16	Orange	Irregular	++	4–12	15–60
RHFS17	Translucent	Lobate	+++	4–12	15–50
RHFS18	Milky white	Filamentous	++	4–12	15–60
RHFS19	Milky white	Irregular	+++	4–12	15–60
RHFS20	Creamy-white	Undulate	++	4–12	15–40
RHFS22	Yellow	Translucent	+++	4–12	15–40
RHFS28	Creamy-white	Circular	++	4–12	15–40

*Anaerobic growth: +:low growth; ++:moderately growth; +++:high growth.

Table S2. 16S rRNA gene-based molecular identity of isolated spore-forming bacteria, their accession numbers, and strain identification is reported.

Bacteria ID	Accession number	Nearest neighbor from NCBI with Accession number	Sequence similarity (%)
RHFS1	MW678576	*Bacillus licheniformis strain QT-98 (MT065812.1)*	98.96
RHFS2	MW678577	*Bacillus mojavensis strain YZJP308 (MN931392.1)*	100.00
RHFS3	MW678578	*Bacillus paralicheniformis strain B34-013 (MK063845.1)*	100.00
RHFS4	MW678579	*Bacillus subtilis subsp. inaquosorum strain YZYR10 (MN931257.1)*	100.00
RHFS5	MW677571	*Bacillus velezensis strain EMP09 (MN062933.1)*	100.00
RHFS6	MW677572	*Bacillus halotolerans strain FUM1 (MK093005.1)*	100.00
RHFS7	MW678580	*Bacillus flexus strain S6c (MT645459.1)*	99.65
RHFS8	MW678581	*Bacillus licheniformis SV12 (LC422787.1)*	99.89
RHFS9	MW677573	*Bacillus velezensis strain XC1 (MT649755.1)*	99.85
RHFS10	MW678582	*Bacillus vallismortis strain CBs8 (MK290421.1)*	100.00
RHFS11	MW678583	*Bacillus velezensis strain GST21(MN809529.1)*	100.00
RHFS12	MW678584	*Bacillus subtilis strain HR02 (MK283755.1)*	100.00
RHFS13	MW678585	*Bacillus tequilensis strain R-QL-48-26(MT078639.1)*	100.00
RHFS14	MW678586	*Bacillus subtilis strain R47 (MH359177.1)*	90.19
RHFS15	MW678587	*Bacillus velezensis strain 1601 (MW242869.1)*	100.00
RHFS16	MW678588	*Bacillus toyonensis strain IBB-TEB3(MT573517.1)*	100.00
RHFS17	MW678589	*Bacillus amyloliquefaciens strain NO10 (MT377854.1)*	100.00
RHFS18	MW678590	*Bacillus amyloliquefaciens strain S8TS (MK729078.1)*	100.00
RHFS19	MW677570	*Bacillus paralicheniformis strain AJVR1(MT459810.1)*	100.00
RHFS20	MW678591	*Bacillus proteoliticus strain 1372 (MT573794.1)*	100.00
RHFS22	MW678592	*Bacillus tequilensis strain CFR01 (MT641220.1)*	100.00
RHFS28	MW678593	*Bacillus cereus strain F3-1-38 (KX350019.1)*	100.00

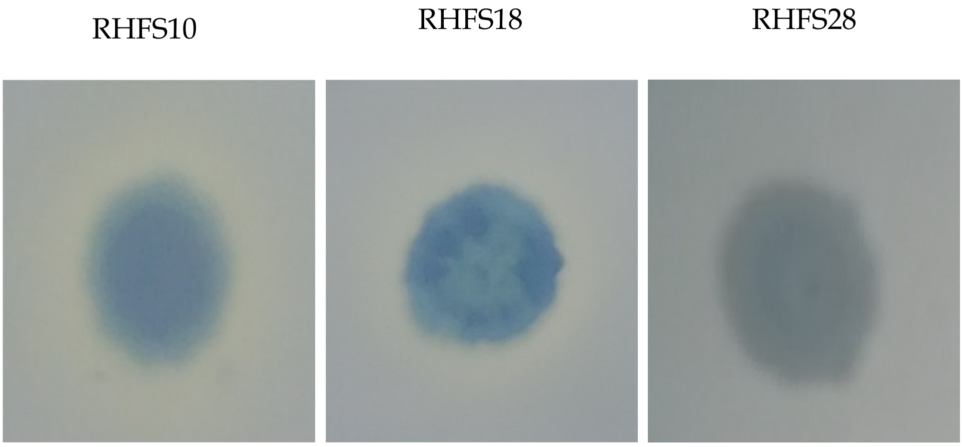

Figure S1. Potential plant growth-promoting traits of selected bacterial isolates. The ability to solubilize inorganic phosphate was assessed by growing the bacterial isolates on the Pikovskaya agar assay [30]. The experiment was performed in triplicate with three independent trials.

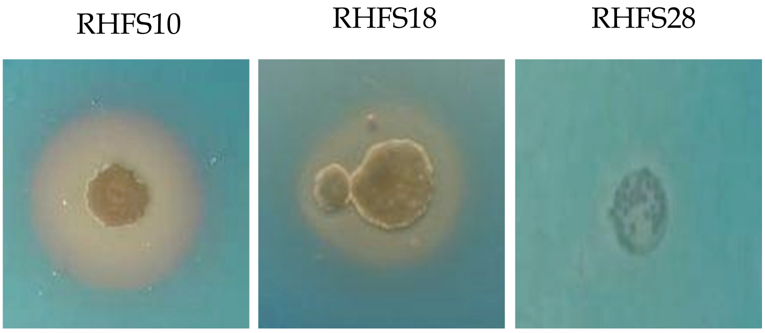

Figure S2. Potential plant growth-promoting traits of selected bacterial isolates. Siderophore production was assessed through O-CAS assay method [31]. The experiment was performed in triplicate with three independent trials.

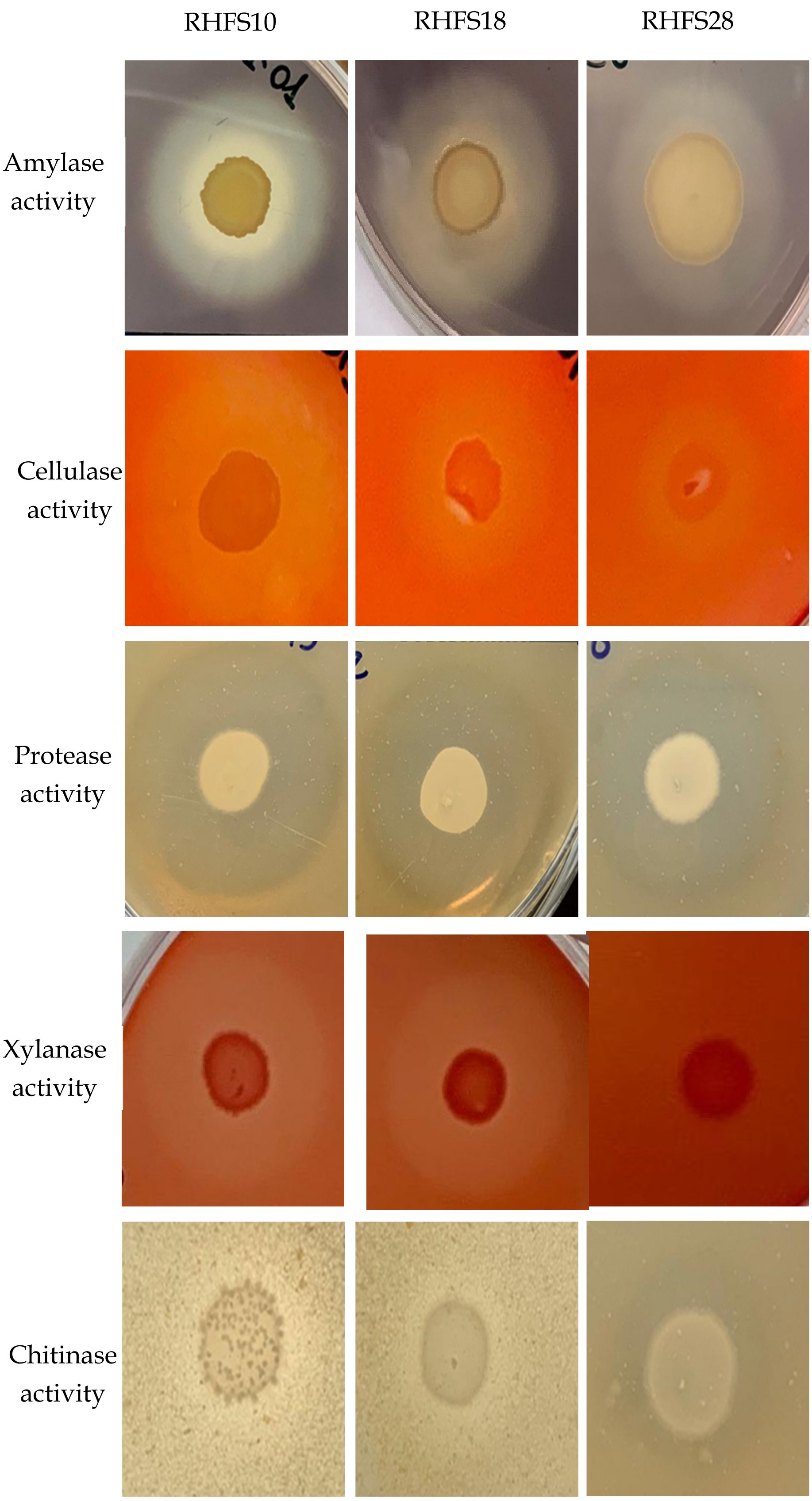

Figure S3. Hydrolytic activities of selected bacterial isolates [33–39]. The experiment was performed in triplicate with three independent trials.

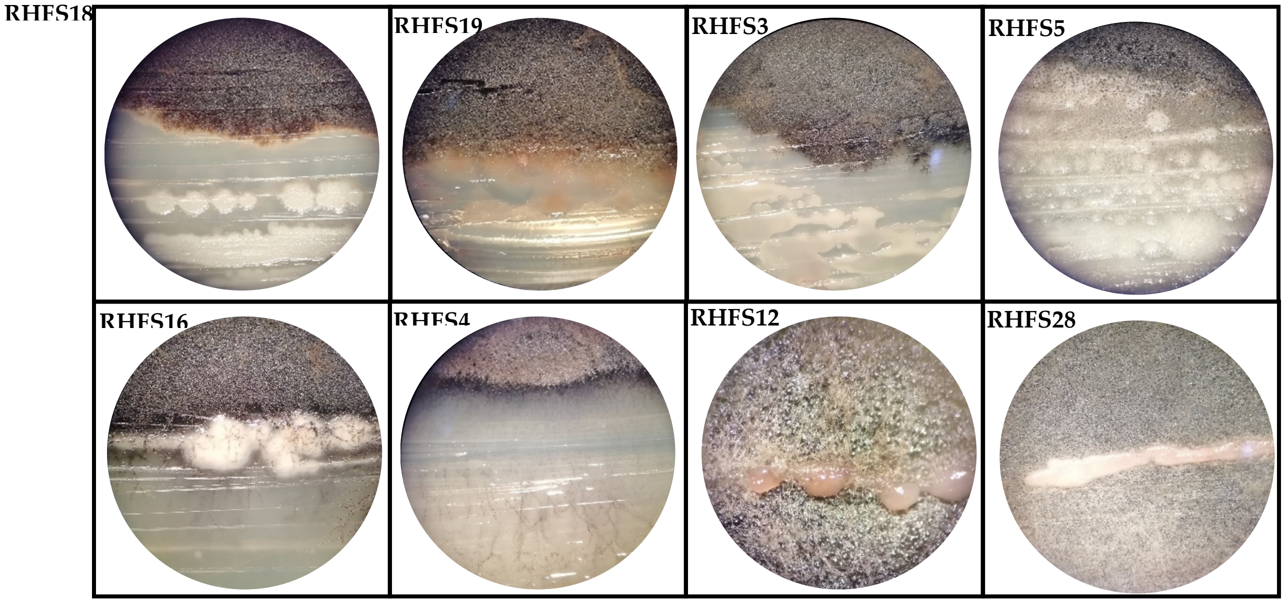

Figure S4. Preliminary dual-culture assay. The figure shows other examples of spore-forming bacteria isolated from the rhizosphere of *J.sabina plants* tested against *M. phaseolina* with antifungal activity (RHFS18, RHFS19, RHFS3, RHFS5, RHFS16, and RHFS4) and examples of bacteria not able to inhibit the fungal growth (RHFS12 and RHFS28). All plates were observed under a stereoscopic microscope (10× magnification) [40].
